# Supplementary material for: Mechanisms governing the pioneering and redistribution capabilities of the non-classical pioneer PU.1
Source: Nat Commun. 2020 Jan 21;11:402. doi: 10.1038/s41467-019-13960-2 (PMC6972792; doi:10.1038/s41467-019-13960-2)
Supplement: Supplementary file 7 — Source data [file 41467_2019_13960_MOESM7_ESM.zip › Source_Data/Figure6/Figure6B_MotifScanOutput/FLI1_mutPU.1/homerResults/motif7.similar.html]

motif7

## Information for motif7

A
C
T
G
A
C
T
G
A
G
C
T
G
A
C
T
G
A
C
T
A
G
T
C
G
T
A
C
A
T
C
G
C
A
G
T
A
C
G
T
A
C
G
T
  
Reverse Opposite:  

T
G
C
A
T
G
C
A
G
T
C
A
T
A
G
C
C
A
T
G
T
C
A
G
C
T
G
A
C
T
G
A
T
C
G
A
T
G
A
C
T
G
A
C
  

|  |  |
| --- | --- |
| p-value: | 1e-158 |
| log p-value: | -3.642e+02 |
| Information Content per bp: | 1.565 |
| Number of Target Sequences with motif | 1001.0 |
| Percentage of Target Sequences with motif | 9.76% |
| Number of Background Sequences with motif | 1149.4 |
| Percentage of Background Sequences with motif | 3.02% |
| Average Position of motif in Targets | 102.7 +/- 54.5bp |
| Average Position of motif in Background | 96.6 +/- 67.5bp |
| Strand Bias (log2 ratio + to - strand density) | -0.1 |
| Multiplicity (# of sites on avg that occur together) | 1.03 |
| Motif File: | file (matrix) reverse opposite |

### Similar de novo motifs found

|  |  |  |  |  |  |  |  |
| --- | --- | --- | --- | --- | --- | --- | --- |
| Rank | Match Score | Redundant Motif | P-value | log P-value | % of Targets | % of Background | Motif file |
| 1 | 0.910 | G C T A G T C A A G T C A T C G A T C G G C T A T C G A T G C A A T C G | 1e-139 | -320.505265 | 10.45% | 3.73% | motif file (matrix) |
| 2 | 0.833 | C G T A C G T A G T C A G T A C C G A T A T C G C G T A C G T A C T G A A T G C | 1e-22 | -50.689984 | 1.57% | 0.54% | motif file (matrix) |
